# Understanding Consumer Financial Trust Across National Levels of Interpersonal Trust †

**DOI:** 10.3390/bs16010062

**Published:** 2025-12-30

**Authors:** Torben Hansen, Claus Varnes

**Affiliations:** Department of Marketing, Copenhagen Business School, Solbjerg Pl. 3, 2000 Frederiksberg, Denmark

**Keywords:** general financial trust, interpersonal trust, consumer behavior, cross-national comparison, structural equation modeling, consumer satisfaction in financial services

## Abstract

Trust plays a pivotal role in consumer decision-making processes, especially in complex areas such as financial services. This study analyzes how general financial trust (GFT)—understood as consumers’ overall perception of the trustworthiness of financial institutions—is influenced by national levels of interpersonal trust (IPT) and how this interaction affects key psychological factors in the relationship between customers and financial service providers. Drawing on theories of cognitive consistency, attribution, and prospects, our results indicate that in national markets where the IPT level is low (vs. high), consumers are (a) more inclined to take GFT into account as a factor that directly influences their anticipated outcome (i.e., expectations) and perceived outcome (i.e., quality and satisfaction) and (b) less inclined to take GFT into account as a contextual moderator of the relationships between expectations, quality, and satisfaction. Our study is based on two surveys with bank customers in Sweden (*n* = 6049) and Spain (*n* = 1050), respectively. In this study, Sweden represented a national market with relatively high-level IPT (i.e., 63.8% of citizens agreed with the statement ‘most people can be trusted’), whereas Spain was a national market with low-level IPT (i.e., 32.8% of citizens agree with the statement ‘most people can be trusted’).

## 1. Introduction

Research has consistently shown that trust is a fundamental psychological factor influencing consumer behavior, especially in situations involving uncertainty, risk, and long-term commitments (Royo-Vela et al., 2024; Van der Cruijsen et al., 2021; Eisingerich & Bell, 2007). In the financial service sector, consumer trust has become an increasingly important area of study, shaped by various market developments and events. The financial crisis, for example, prompted closer scrutiny of trust levels within the industry (Hansen, 2012a, 2012b, 2024). More recently, the collapse of Silicon Valley Bank and failures among other banking institutions have further intensified concerns about trust in banks globally (Partington et al., 2023).

Trust not only relates to consumer trust in individual companies (i.e., individual financial trust, IFT), but also to the more general and broader business context in which consumers may plan and carry out their activities (i.e., general financial trust, GFT). A number of studies have empirically considered consumer GFT in a market context using either general contextual approaches (i.e., comparing one market situation with another market situation at different points in time) (e.g., Hansen, 2014) or more specific contextual approaches (i.e., using GFT as a direct or moderating construct in analyzing customer–seller interactions at one point in time) (e.g., Van der Cruijsen et al., 2021; Grayson et al., 2008). Clearly, none of these studies have considered how variations in national levels of interpersonal trust (IPT) can impact the strength and direction of the GFT–customer relationship link. IPT can be conceptualized as the general tendency of an individual to trust others (Van der Cruijsen et al., 2023; Johnson-George & Swap, 1982). This is unfortunate since taking national levels of IPT into account is highly relevant from a practical point of view, and research suggests that national markets may indeed differ in their cultural and economic factors (Haberstroh et al., 2018), which in turn may affect consumers’ choices, intentions, and behavior (Jiao et al., 2018). Indeed, “interpersonal trust is a basic feature of all social situations that demand cooperation and interdependence” (Johnson-George & Swap, 1982, p. 1306), such as customer–seller financial relationships. 

This study contributes to the behavioral sciences by proposing and demonstrating that national levels of IPT may moderate the influence of GFT on these key psychological outcomes in consumer–bank relationships: expectations, perceived service quality, and satisfaction. The remainder of this paper is organized as follows: First, the theoretical framework and hypotheses are introduced, followed by a review of the methods used to test the hypotheses. Next, the results are presented. Finally, the implications of the findings are discussed, and suggestions for future research are provided.

## 2. Conceptual Model and Research Hypotheses

**Figure 1:**
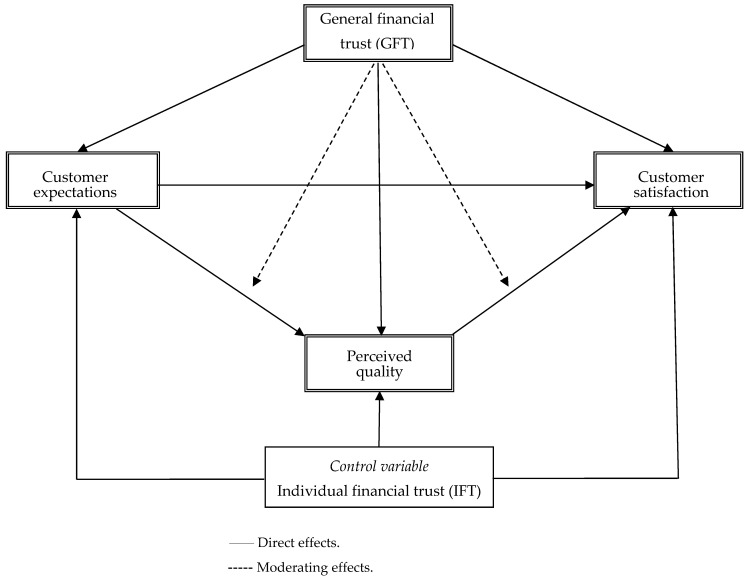
The conceptual baseline model.

This section consists of two main parts. In the first part, a conceptual baseline (non-hypothesized) model is introduced (Figure 1), alongside a discussion of its theoretical underpinnings. The second part hypothesizes how these relationships may differ according to varying levels of IPT.

### 2.1. Baseline Model

Our baseline model focuses specifically on three relationship variables: service expectations, service quality, and customer satisfaction. First, the (positive) relationships between these variables have received much attention and are all well-established in the literature (e.g., Tedja et al., 2024; Habel et al., 2016; EPSI Research Services, 2013). Second, these variables are especially relevant in a trust context and when dealing with complex products, such as financial services, where the information is perceived as complex and/or associated with uncertainty (Van der Cruijsen et al., 2021). Many consumers undoubtedly possess highly limited knowledge about financial products and often regard them as complex (e.g., Chen et al., 2022), which may boost the demand for trust and the need to develop product expectations.

Our baseline model also includes GFT. Although previous research has investigated its influence on customer expectations, perceived quality, and customer satisfaction, the evidence remains limited. However, we expect GFT to positively influence all three variables. When the GFT level is low, not every service provider can be trusted to deliver satisfying services, increasing the risk of poor outcomes. However, many consumers possess highly limited knowledge about financial products (e.g., Lee & Hanna, 2022; O’Connor, 2019). Thus, the consumer is at risk of her/his interests not being properly protected. In such incidents, previous research suggests that in order to maintain self-confidence and to avoid cognitive dissonance the consumer will assign external blame for perceived negative experiences (Gotlieb, 2009), which may reduce customer expectations, perceived quality, and customer satisfaction.

*Conceptualization of baseline model constructs.* A review of the perceived quality and customer satisfaction literature unveils an ambiguous array of definitions of customer expectations. We find that the following definitions serve our study purposes. *Customer expectations*. In the present study, customer expectations represent the accumulated service-related expectations the customer has developed based on prior experience with the bank’s service offerings (Anderson & Fornell, 2000). *Perceived quality.* In line with Fornell et al. (1996) and Johnson et al. (2002), perceived quality is conceptualized as the customer’s evaluation of recent experiences concerning the quality of their acquired services. *Customer satisfaction*. Customer satisfaction research has focused on two different types of evaluations: transaction-specific satisfaction and cumulative satisfaction (Johnson et al., 2002). Similar to previous customer relationship and service-related research (Nanda & Banerjee, 2021; Guo et al., 2013), customer satisfaction is in the present study conceptualized as an overall, cumulative consumer evaluation of her/his relationship with a financial service provider. Cumulative satisfaction recognizes that customers may rely on their entire experience with a financial service provider when evaluating its performance (Dimitriades, 2006).

*General financial trust (GFT)*. While individual financial trust (i.e., IFT) has been extensively investigated within the consumer trust literature (e.g., Burke & Hung, 2021; Morgan & Hunt, 1994), there is still not a more throughout understanding of GFT. Consistent with prior research, we conceptualize GFT as consumers’ expectation that companies within a certain business type are generally dependable and can be relied on to deliver on their promises. This definition is consistent with previous research suggesting that informal GFT (or generalized trust) is a generalized expectancy that a group can be relied upon (Rotter, 1980; Siegrist et al., 2005). Hence, while IFT pertains to trust in a specific bank, GFT refers to trust in companies within a certain business type (such as banks) as a whole.

*Control variable.* Since our focus in this study is on GFT, IFT is included as a control variable in the model (Greene, 2000). While a large body of research about the concept of IFT exists, with different points of views being advocated, we adapt the often-cited definition proposed by Sirdeshmukh et al. (2002) and conceptualize *IFT* as ‘‘the expectation held by the consumer that the service provider [i.e., the specific individual bank] is dependable and can be relied on to deliver on its promises’’ (p. 17).

### 2.2. Hypothesis Development

We argue that the anticipated positive influence of GFT on customer expectations, perceived quality, and customer satisfaction will be larger in national markets where IPT is at a low level than in national markets where IPT is at a high level. The primary mechanism underlying this argument is heuristic use under uncertainty. Previous research suggests that GFT may operate as a heuristics (Siegrist et al., 2005; Sjöberg, 2001), which can be regarded as ‘inferential rules of thumb’ (Allison et al., 1990). This is because consumers may rely on GFT to reduce the complexity they are faced with when choosing among various services and when evaluating their outcome (Siegrist & Cvetkovich, 2000). The cognitive consistency theory (Festinger, 1957; Heider, 1946, 1979; Newcomb, 1953; Osgood & Tannenbaum, 1955) further suggests that consumers seek mental justification to avoid cognitive dissonance (Todd & Gigerenzer, 2003). Consumers may easily gain mental justification when the IPT level is high, but may experience a mental imbalance when the IPT level is low since their choices are less easily confirmed. Under such conditions, GFT may function as a stabilizing heuristic, signaling systemic reliability, and thereby reducing dissonance. This means that in contexts with low-level IPT, GFT may constitute a more prominent predictor of expectations and satisfaction. This aligns with threat–rigidity theory, which suggests that individuals under perceived threat or uncertainty depend on simple, risk-reducing cues (Jeong et al., 2023). Thus, when consumers feel vulnerable to market failure, GFT functions as a stabilizing heuristic, guiding expectations and satisfaction.

The attribution theory (Weiner, 1986, 2000; Tomlinson & Mayer, 2009) provides additional support for this reasoning. In high-level IPT environments, consumers may attribute positive outcomes to stable societal factors, thereby reducing their reliance on generalized heuristics such as GFT. In contrast, when the IPT level is low, consumers may take situational cues more closely into account using GFT as an external attribution that compensates for the perceived relational risk. This mechanism suggests that while GFT primarily serves as a heuristic, it also serves as an explanatory anchor under conditions of low-level IPT.

Based on the above reasoning, it can therefore be expected that in markets where IPT is at a low level (i.e., indicating risk), consumers should be *more* inclined to take GFT into account when determining their anticipated or perceived outcome. Hence, increases in GFT should be expected to be especially useful in national markets where IPT at on a low level. In sum, the following research hypotheses are proposed.

**H1:**  *The positive influence of GFT on customer expectations is larger when the IPT level is low compared to high.*

**H2:**  *The positive influence of GFT on perceived quality is larger when the IPT level is low compared to high.*

**H3:**  *The positive influence of GFT on customer satisfaction is larger when the IPT level is low compared to high.*

We propose that GFT moderates the relationships between expectations, perceived quality, and satisfaction, and that the strength of these moderations is influenced by IPT. The primary mechanism underlying these suggestions is the attribution theory (Weiner, 1986, 2000; Tomlinson & Mayer, 2009). Several insightful studies have investigated trust using an attribution theory approach. As an overall conclusion, these studies indicate that trust in a relationship (i.e., IFT) is enhanced to the extent that the other’s trustworthiness can be ascribed to factors that are internal to the trustee, rather than situationally driven (e.g., Hansen, 2012a, 2012b; Tomlinson & Mayer, 2009). However, in this study, we contribute to previous research by arguing that the moderating effects suggested by previous research will be affected by IPT such that negative moderating effects are more likely to occur in national markets where IPT is at a high absolute level compared to national markets where IPT is at a low absolute level. When the IPT level is low, it points to the existence of financial system failures, which means that the consumer’s interests are not being properly served. When faced with such circumstances, the consumer choice theory suggests that consumers are generally more likely to thoroughly evaluate the more specific consequences both of choosing one alternative and of foregoing the other (Shiu et al., 2011). Hence, the consumer could be expected to pay more attention to her/his expectations/evaluations when determining the cause for a specific relationship outcome, thereby reducing the reliance on trust (Dixon & Wilkinson, 1989). Hence, in markets where IPT is at a low level, consumers should be *less* inclined to take into account GFT as the *cause* of their perceived outcome (i.e., in the present context, perceived quality and customer satisfaction) than in markets where IPT is at a high level.

The prospect theory (Kahneman & Tversky, 1979) provides additional support for this reasoning by emphasizing that consumers tend to assign greater psychological weight to losses under uncertainty. In countries with low-level IPT, financial decisions may be perceived as more uncertain and associated with higher risk. In such cases, GFT may function as a form of mental safety net that helps consumers navigate a market characterized by uncertainty and maintain coherence among key relationship variables such as expectations, perceived quality, and satisfaction. Conversely, in high-level IPT contexts, systemic trust reduces uncertainty, diminishing the reliance on GFT and potentially weakening its moderating role. This substitution effect weakens—and may even negatively moderate—the influence of expectations on quality and of quality on satisfaction because consumers rely more on trust signals than on detailed service assessments.

In sum, the following hypotheses are proposed.

**H4:**  *The influence of customer expectations on perceived quality is more likely to be negatively moderated by GFT when the IPT level is high compared to low.*

**H5:**  *The influence of perceived quality on customer satisfaction is more likely to be negatively moderated by GFT when the IPT level is high compared to low.*

## 3. Methodology

### 3.1. Data Collection

Two countries were selected for this study—Sweden and Spain—with Sweden representing a national market with high-level IPT (i.e., 63.8% of citizens agree with the statement ‘most people can be trusted’) and Spain representing a national market with low-level IPT (i.e., 32.8% of citizens agree with the statement ‘most people can be trusted’) (Ortiz-Ospina et al., 2023). Sweden and Spain were selected because they exhibit a substantial and well-documented gap in IPT. The IPT difference of more than 30 percentage points aligns with prior research (e.g., Zak & Knack, 2001; Kumar & Pansari, 2016) and is consistent with thresholds typically regarded as meaningful for categorical contrasts in national trust variables (Ward et al., 2014). 

In this study, we consider IPT as a cultural factor that is located at the macro level rather than as an individual variable. Thus, IPT is operationalized on the basis of nationally representative data from Our World in Data (Ortiz-Ospina et al., 2023). Here, the proportion of citizens who agree with the statement ‘most people can be trusted’ is used as an indicator of IPT. The purpose of this is to obtain an indication of the levels of trust in the two countries (Sweden vs. Spain) in order to analyze how these levels could affect the importance of GFT in relationships between consumers and banks. It is clear that more individual-based measurements could have added further details to this study, but our present focus here is to explore the structural differences between national markets.

In each country, the data were collected by telephone interviews with bank customers conducted by a professional market research agency as a part of the pan European EPSI (Extended Performance Satisfaction Index) customer satisfaction study. Telephone interviews were chosen to maintain consistency with the EPSI framework, which prioritizes interviewer-assisted data collection for complex topics like financial trust. This approach may reduce non-response bias and clarify ambiguous questions more compared to self-administered online surveys (e.g., Fowler, 2002). The largest banks were selected, such that coverage included the majority of the bank market. For each bank, at least 200 customers were interviewed, and in total 6049 interviews with Swedish bank customers and 1050 interviews with Spanish bank customers were conducted and used for this study.

### 3.2. Measurements

The measures used in this study are those developed for the pan European EPSI customer satisfaction model and analysis. Customer expectations were measured by four items: products (services) offered by the bank, added functions offered, personal service and advice, and reliable and accurate service. Perceived quality was measured by four items relating to both product and service quality: the banking products offered, added functions offered, advice and information given by the personnel of the bank, and politeness and friendliness of personnel. Customer satisfaction was measured by three items: overall satisfaction, comparison with an ideal bank, and fulfilment of expectations. These measurements of customer satisfaction are commonly used in research concerning customer–seller relationships (e.g., Auh & Johnson, 2005; Martensen & Grønholdt, 2010). *GFT* and *IFT* were measured by two items concerning the customers’ perceived level of trust in the banking sector and trust in their current bank, respectively (e.g., Pérez & Descals, 1999; Hansen, 2012a). All items were measured on 10-point scales.

## 4. Results

Confirmatory factor analysis (CFA) was conducted on the four latent variables, with each indicator specified to load on its hypothesized latent factor. The measurement model for each sample was examined individually to take into account the difference in sample size (Table 1).

The measurement models yields the following chi-square values—Sweden: 1970.74 (d.f. = 41, *p* < 0.01); Spain: 690.98 (d.f. = 41, *p* < 0.01)—suggesting a lack of absolute model fit. However, since the chi-square test is highly sensitive to sample size, other fit measures are given greater prominence in evaluating model fit (e.g., Ye et al., 2007). The comparative fit index (CFI) (Sweden = 0.96; Spain = 0.94) and the normed fit index (NFI) (Sweden = 0.96; Spain = 0.93) both suggest an acceptable degree of fit of the measurement model (Bagozzi & Yi, 1988). The testing of path differences between samples assumes measurement invariance, meaning that the construct measures are assumed to be invariant across each of the two levels. A chi-square difference test between the unconstrained model and a model where the measurement weights were constrained to be equal across groups suggest that the applied measures are invariant across groups: Δχ^2^ = 13.39, Δd.f. = 8, *p* = 0.10.

All composite reliabilities exceed 0.80, indicating good reliability of the measured constructs. Also, the extracted variance is greater than 0.60 for all the latent constructs, which satisfies the threshold value recommended by Fornell and Larcker (1981). An examination of Table 2 shows that the extracted variance for each of the constructs exceeds the squared correlation, except for ‘customer satisfaction’ with respect to its correlation with ‘perceived quality’ (Swedish sample) (variance, satisfaction = 0.65 *<* squared correlation satisfaction-quality = 0.73), although the latter is below the suggested threshold of 0.85 (Frambach et al., 2003). Also, as a relationship between quality and satisfaction is expected in the conceptual model, the relatively high correlation should not be regarded as a serious violation of discriminant validity. In order, to further inspect the distribution of measurement items across constructs, a three-dimension principal component analysis (PCA) with oblimin rotation (Kaiser normalization) was conducted for the Swedish and Spanish samples. For both the samples, the item distribution patterns suggested that the applied items constituted viable representations of their specified underlying constructs.

As previous research (e.g., Grayson et al., 2008) has detected a significant correlation between GFT and IFT, we also specified two additional models for both the samples, one with GFT as an endogenous variable (and all other variables as exogenous) and one with IFT as an endogenous variable (and all other variables as exogenous). In both the cases, the variance inflation factors (VIFs) were below five, indicating that no serious multicollinearity problems were present (e.g., Hair et al., 2019). This is consistent with the finding that the shared variance between constructs was only 0.01 (Sweden)/0.08 (Spain) (Table 2), corresponding to the following correlations *r* = 0.07, *p* < 0.01 (Sweden), and *r* = 0.28, *p* < 0.01 (Spain). Although the correlations were both significant, their size can be considered less substantial (Cohen, 1988).

**Figure 2:**
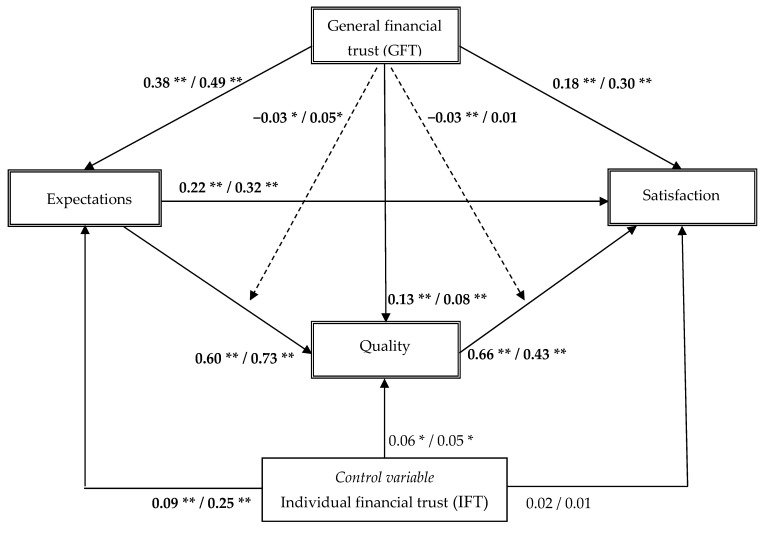
The estimated model. Notes: Model fit: (χ^2^ = 5663.69, d.f. = 348, *p* < 0.01; CFI = 0.95; NFI = 0.95; Hoelter(.05) = 493). Coefficients marked in bold are statistically different (α = 0.05). Order of standardized coefficients displayed: Swedish sample (high-level IPT)/Spanish sample (low-level IPT). **: *p*-value < 0.01; *: *p*-value < 0.05.

The conceptual model (Figure 1) was analyzed using structural equation modeling (SEM), and the model was estimated and tested using a covariance-based SEM method using SPSS AMOS 28. Initially, an estimation of the model for each of the two samples separately showed a reasonable model fit in both the incidents (Swedish sample: χ^2^ = 4249.01, d.f. = 174, CFI = 0.96, NFI = 0.96; Spanish sample: χ^2^ = 1371.22, d.f. = 174, CFI = 0.94, NFI = 0.93). The hypothesized model was then fitted simultaneously to the Swedish and Spanish samples using multiple-group latent variable structural equation modeling (SEM) analysis. In the SEM model, the interaction effects (i.e., GFT × expectations and GFT × quality, respectively) were formed using the residual-centering (i.e., orthogonalizing), two-step procedure recommended by Little et al. (2006).The model fits the data reasonably well (χ^2^ = 5663.69, d.f. = 348, *p* < 0.01; CFI = 0.95; NFI = 0.95; Hoelter (0.05) = 493). Figure 2 displays the multiple-group SEM results.

As expected, the results suggest that the positive influence of GFT on expectations was larger in Spain (β = 0.49, *p* < 0.01) than in Sweden (β = 0.38, *p* < 0.01). The difference between the coefficients was significant (Δχ^2^ = 45.09, Δd.f. = 1; *p* < 0.01). Hence, H1 was supported in this study. Rejecting H2, the influence of GFT on quality was smaller in Spain than in Sweden (Spain; β = 0.08, *p* < 0.01; Sweden: β = 0.13, *p* < 0.01; Δχ^2^ = 6.08, Δd.f. = 1; *p* = 0.01). Supporting H3, the influence of GFT on customer satisfaction was larger in Spain (β = 0.30, *p* < 0.01) than in Sweden (β = 0.18, *p* < 0.01) (Δχ^2^ = 42.10, Δd.f. = 1; *p* < 0.01). In addition, the results suggest that the influence of customer expectations on perceived quality was negatively moderated by GFT in Sweden (β = −0.03, *p* = 0.02) and positively moderated by GFT in Spain (β = 0.05, *p* = 0.02) (Δχ^2^ = 14.01; Δd.f. = 1, *p* < 0.01). Hence, H4 was supported. Consistent with our expectations, the influence of quality on customer satisfaction was negatively moderated by GFT in Sweden (β = −0.03, *p* < 0.01), whereas no moderating effect was detected for Spain (β = 0.01, *p* = 0.87) (Δχ^2^ = 6.00, Δd.f. = 1; *p* = 0.01). Hence, H5 was also supported in this study.

## 5. Discussion

This study provides evidence that GFT has direct positive effects on customer expectations, perceived quality, and satisfaction, respectively, as well as moderating effects on the relationship between expectations and perceived quality and the relationship between perceived quality and customer satisfaction. Our results reveal interesting differences between impacts of GFT in two national markets, which differ according to IPT (low vs. high level). The findings provide strong empirical evidence for the developed conceptual model and four of the five proposed hypotheses regarding low vs. high absolute levels of GFT in national markets were supported.

### 5.1. Implications for Theory

To our knowledge, this study is the first to investigate how relations between GFT and customer–seller relationship variables may vary across levels of IPT in different national markets. This is an important extension of previous research because studies based solely on one national marketplace may face the risk of under- or overstating the influence of GFT on relationship variables (Hansen, 2014). To facilitate our study, we developed a theoretical framework, which specifies the relationships between GFT and customer expectations, perceived quality, and satisfaction, respectively, and which includes IFT as a control variable. Our findings suggest that GFT can act very differently depending upon (a) whether GFT is viewed as a factor that directly influences anticipated (i.e., expectations) and perceived outcomes (i.e., quality and satisfaction); (b) whether GFT is seen as a contextual effect moderating relationships between expectations, perceived quality, and satisfaction; and/or (c) whether the functioning of GFT is studied in national markets with a low (vs high) level of IPT.

In both the investigated national markets (i.e., Sweden and Spain), we found that GFT was positively related to expectations, perceived quality, and satisfaction, respectively. Hence, our results confirm the suggestions put forward by previous research that when faced with complex decisions, people are likely to seek guidance from available heuristics (i.e., GFT) when seeking to assess the possible outcome of their decision (Tedja et al., 2024, Grayson et al., 2008; Hansen, 2012a). However, as an extension of previous consumer trust research, we also found that the effect of GFT on anticipated outcomes is likely to be influenced by IPT. Specifically, we found that when IPT is at a low level, consumers seems to be *more* inclined to take into account GFT as an indicator of their expectations and as a variable influencing their level of satisfaction. Hence, this study provides further evidence for the usefulness of incorporating GFT as a contextual factor when investigating customer–seller relationships. Contrary to our expectations, we also found that in markets where the IPT level is low, consumers were less inclined to use GFT an indicator of quality. A possible explanation for this result could be that assessments of quality include both experiential and relational aspects (Tran, 2020). These may be more prominent in environments with high-level IPT, where trust in itself may reinforce positive interpretations of service encounters. Conversely, consumers in low-level IPT societies may be more vigilant and adopt a more evidence-based approach, more carefully evaluating service quality characteristics—such as employee behavior, responsiveness, and product features—and less likely to rely on broad trust signals. 

However, by showing that the positive influence of GFT on customer expectations and satisfaction is higher in when IPT is low (vs. high)-level, this study establishes both GFT and IPT as key characteristics in understanding consumer financial relationship behavior. On a similar vein, and consistent with our expectations, we found that when the IPT level was high, GFT negatively moderated the relationships between expectations and quality and between quality and satisfaction, whereas positive (the expectation–quality relationship) and no moderation (the quality–satisfaction relationship), respectively, were found when the IPT level was low. Our study also included IFT as a control variable.

Although not specifically hypothesized, we found that the influence of IFT on expectations, quality, and satisfaction showed a pattern almost similar to the pattern exhibited by GFT (although the IFT–satisfaction path was not significant in both the countries). Similar to GFT, IFT had a more positive relationship with expectations in Spain (low-level IPT) than in Sweden (high-level IPT). The rather similar patterns of GFT and IFT provides support for the institutional theory (Scott, 2005; Meyer & Rowan, 1977; DiMaggio & Powell, 1983), which suggests that if trust is common within a business domain, it encourages the development of trust in other domains. The patterns of GFT and IFT are also consistent with previous research (e.g., Grayson et al., 2008), which has detected a significant correlation between GFT and IFT.

### 5.2. Implications for Practice

Our results have important implications for both international financial service managers and financial authorities. Managers operating in national markets where the IPT level is low should be especially concerned that a decreasing level of GFT may have a strong direct negative influence on expectations and satisfaction. On the other hand, with higher levels of IPT, managers in such markets may benefit from an improved relationship between GFT and these relationship variables. When considering how to approach their customers and the amount of resources (staff resources, market campaigns, etc.) needed in order to develop trustful relationships with customers, financial services managers should take into account the trustworthiness of other service providers as detailed in the following text.

*In national markets where the IPT level is high*, the positive influence of consumer expectations on perceived quality and of perceived quality on customer satisfaction decreases with a further increase in GFT. However, this may be compensated by the positive relationships between GFT and each of the three relationship variables, although the degree of compensation is higher in national markets where the IPT level is low (i.e., GFT has a stronger relationship with expectations and satisfaction in such markets). Since expectations and perceived quality are less-important determinants of satisfaction when the GFT level is high, financial service managers may instead wish to consider a combination of (a) directing a larger amount of their resources at attracting new customers (because the IPT level is high, consumers should be expected to be less skeptical when exposed to financial market campaigns from alternate service providers) and (b) providing existing customers with favorable offerings in order to make them more reluctant to market communication and offers from competing financial service providers.

*In national markets where the IPT level is low*, financial service providers will gain a higher level of perceived quality from developing positive expectations when GFT is increasing. Hence, in such a situation, managers should carefully outline all the positive outcomes (e.g., reliable and accurate service, personal service, and advice, among others) that customers may expect from their services and should also consider developing additional services (e.g., favorable savings accounts, financial budgeting services, and the like) specifically aimed at improving customers’ expectations. On the other hand, when GFT is decreasing, financial managers should consider investing additional resources in more direct improvements of perceived quality (e.g., training personnel to show more polite and friendly attitudes and show a high level of commitment in individual customers, among others).

*In both types of national market*, the revealed positive relationships between GFT and customer expectations, perceived quality, and satisfaction suggest that individual banks should be concerned with improving GFT. However, because of market competition and scarce resources, some bank manages may feel tempted only to focus on improving expectations, perceived quality, and/or satisfaction since all service providers benefit when the GFT level is high (Carlin et al., 2009; Hansen, 2012b; Williamson, 1993). That is, a free-riding potential may exist. Because it is in the interest of societies that their citizens have trust in the financial system (Buriak et al., 2019; Sapienza & Zingales, 2012; Earle, 2009), this underscores the importance of developing well-functioning financial regulations, which ensures that GFT is not undermined by ‘free-riding’ service providers.

### 5.3. Limitations and Future Research

The respondents were approached via telephone interviews; they may behave differently when engaging in specific settings. Thus, although a survey is generally accepted as a means of data collection, there is little control over the contextual setting and over the response behavior of consumers. A limitation of this study is that only a single item was used to measure GFT and IFT, respectively. Although this approach is consistent with the EPSI framework, which uses single-item indicators to ensure comparability across large, cross-national studies, the use of single-item measures for GFT and IFT could have an impact on both direct and interaction effects in the estimated structural model. While single-item measures imply conceptual simplicity and high face validity, they are more vulnerable to random measurement error compared to multi item scales. This may attenuate the estimated path coefficients, leading to conservative estimates of direct effects (Diamantopoulos et al., 2012). This is also critical in relation to interaction effects—such as those modeled between GFT and expectations or perceived quality—as these are typically harder to detect and highly sensitive to measurement error. Measurement error in either interacting variable inflates residual variance and reduces statistical power, meaning that the moderating influences reported in this study may represent lower-bound estimates of the true effects (McClelland & Judd, 1993). Although single-item measures can be useful for generalized trust constructs, as has been shown in previous research (e.g., Rotter, 1980; Siegrist et al., 2005) due to their conceptual simplicity and high face validity, we recommend that future research employ multi item scales for GFT and IFT to enhance reliability and reduce error variance. Additionally, testing measurement invariance across cultural contexts would strengthen the robustness of cross-national comparisons.

Another limitation concerns the design itself as a two-country study. Although Sweden and Spain are specifically selected to represent contexts with high- and low-level IPT, respectively, the design itself limits the generalizability of this study. In addition, the design does not take into account possible variations within the region or alternative country-specific factors such as economic climate, regulatory trust, financial literacy, and cultural norms. To address these aspects in more detail, future research should use data sets from multiple countries or experimental manipulations of IPT in order to validate the moderating role of interpersonal trust and rule out potential confounders. In this study, IPT was conceptualized as a macro-level cultural variable. While this approach aligns with prior cross-cultural research (e.g., Jing et al., 2021), it does not capture individual-level variations in interpersonal trust. Future studies may wish to integrate both macro- and micro-level measures to provide a more nuanced understanding of how personal trust may interact with consumer–bank relationships.

Also, this study used perceptive measures, which could be threatened by biased responses, and builds on the difference in IPT between Sweden and Spain. Although the IPT difference between the two countries is well-established, future research could further examine this issue by manipulating IPT in an experimental setting. Moreover, the detected effects may not generalize to all contexts. Indeed, the influence of GFT on the endogenous variables may vary according to other market circumstances than IPT such as market transparency or specific national regulatory environments. This is because minimal transparency may increase consumer demand for trust, while strong regulatory frameworks might mitigate trust deficits (Zak & Knack, 2001; Tschannen-Moran & Hoy, 2000). However, all the constructs examined in this study are generalizable across financial service businesses, and it is likely that similar effects would be found irrespective of the particular country and/or business being investigated. For example, expectations, perceived quality, and satisfaction are also highly relevant for other financial industries and for other business types. Moreover, the consistency of the findings with the theoretical model suggests that the findings will be similar in other financial services contexts (Guo et al., 2013). Indeed, the theoretical underpinnings regarding the interplay between expectations, perceived quality, GFT, and satisfaction may also hold true for other industries, such as the food market, which also can be characterized by perceived market complexity and demand for trust (e.g., Hansen & Thomsen, 2013).

## Figures and Tables

**Table 1:** Confirmatory factor analyses results.

Construct/Measures	Standardized Factor Loading (Sw./Sp.)	Critical Ratio (χ^2^) (Sw./Sp.)	Composite Reliability (Sw./Sp.)	Extracted Variance (Sw./Sp.)
Customer expectations			0.91/0.92	0.71/0.73
1. Products offered	0.88/0.89	-/-		
2. Personal service and advice	0.83/0.82	82.09/64.69		
3. Reliable and accurate service	0.82/0.90	74.16/35.51		
4. Added functions offered	0.84/0.81	77.07/30.73		
Perceived quality			0.92/0.92	0.74/0.74
5. The personnel’s commitment	0.83/0.89	-/-		
6. Politeness and friendliness of the personnel	0.85/0.78	80.15/32.56		
7. Advice and information given by the personnel	0.89/0.90	86.42/43.29		
8. Banking products offered	0.86/0.87	82.23/40.45		
Customer satisfaction			0.85/0.88	0.65/0.70
9. Overall satisfaction	0.82/0.81	-/-		
10. Fulfilment of expectations	0.78/0.85	65.17/30.91		
11. Close to “ideal” bank	0.81/0.85	64.73/30.85		

Notes: One item for each construct was set to 1. Sw: Sweden; Sp: Spain. General financial trust (GFT) and individual financial trust (IFT) were both measured by a single item, and therefore not included in the confirmatory factor analysis. GFT: Consumers’ expectation that companies within a certain business type are generally dependable and can be relied on to deliver on their promises (Siegrist et al., 2005). IFT: The expectation held by the consumer that the service provider [i.e., the specific individual bank] is dependable and can be relied on to deliver on its promises (Sirdeshmukh et al., 2002).

**Table 2:** Descriptive statistics and discriminant validity of constructs.

	1	2	3	4	5
Construct	Sw./Sp.	Sw./Sp.	Sw./Sp.	Sw./Sp.	Sw./Sp.
1. Customer expectations	0.71/0.73				
2. Perceived quality	0.43/0.64	0.74/0.74			
3. Customer satisfaction	0.52/0.66	0.73/0.67	0.65/0.70		
4. General financial trust (GFT)	0.15/0.28	0.13/0.22	0.25/0.45	n.a.	
5. Individual financial trust (IFT)	0.01/0.13	0.02/0.11	0.02/0.20	0.01/0.08	n.a.
Mean	8.01/6.51	8.24/6.74	7.58/5.88	6.36/4.58	7.57/6.39
SD	1.55/2.07	1.52/2.01	1.55/2.15	1.95/2.49	1.95/2.66

Notes: Diagonals represent average amount of extracted variance for each construct. Non-diagonals represent the shared variance between constructs (calculated as the squares of correlations between constructs). Customer expectations, perceived quality, and satisfaction were measured on 10-point scales ranging from 1 = ’very low’ to 10 = ’very high’. Averaged means are reported for expectations, perceived quality, and satisfaction. Sw: Sweden, high-level IPT; Sp: Spain, low-level IPT. n.a.: General financial trust (GFT) and individual financial trust (IFT) were measured by a single item; therefore, extracted variance could not be computed.

## Data Availability

The original data presented in this study are openly available on the Open Science Framework (OSF) at https://osf.io/qv75z/files/q8gx3 (accessed on 11 November 2025).
